# Body Ownership of Anatomically Implausible Hands in Virtual Reality

**DOI:** 10.3389/fnhum.2021.713931

**Published:** 2021-11-03

**Authors:** Or Yizhar, Jonathan Giron, Mohr Wenger, Debbie Chetrit, Gilad Ostrin, Doron Friedman, Amir Amedi

**Affiliations:** ^1^Department of Cognitive and Brain Sciences, The Hebrew University of Jerusalem, Jerusalem, Israel; ^2^Baruch Ivcher School of Psychology, Interdisciplinary Center Herzliya, Herzliya, Israel; ^3^Innovation Center, Interdisciplinary Center Herzliya, Herzliya, Israel; ^4^Sammy Ofer School of Communications, Interdisciplinary Center Herzliya, Herzliya, Israel; ^5^The Ruth & Meir Rosental Brain Imaging Center, Interdisciplinary Center Herzliya, Herzliya, Israel

**Keywords:** body ownership, virtual reality, body representation, visuomotor interaction, anatomical plausibility, volition, immersive virtual reality

## Abstract

Manipulating sensory and motor cues can cause an illusionary perception of ownership of a fake body part. Presumably, the illusion can work as long as the false body part’s position and appearance are anatomically plausible. Here, we introduce an illusion that challenges past assumptions on body ownership. We used virtual reality to switch and mirror participants’ views of their hands. When a participant moves their physical hand, they see the incongruent virtual hand moving. The result is an anatomically implausible configuration of the fake hand. Despite the hand switch, participants reported significant body ownership sensations over the virtual hands. In the first between-group experiment, we found that the strength of body ownership over the incongruent hands was similar to that of congruent hands. Whereas, in the second within-group experiment, anatomical incongruency significantly decreased body ownership. Still, participants reported significant body ownership sensations of the switched hands. Curiously, we found that perceived levels of agency mediate the effect of anatomical congruency on body ownership. These findings offer a fresh perspective on the relationship between anatomical plausibility and assumed body ownership. We propose that goal-directed and purposeful actions can override anatomical plausibility constraints and discuss this in the context of the immersive properties of virtual reality.

## Introduction

Our body is the source of our experienced sensations and the target of our voluntary actions. Its character is possessive, and we perceive it as our own through self-attribution (Gallagher, 2000; Tsakiris et al., 2006). This phenomenon, termed body ownership, can extend beyond our physical self. For example, synchronous stroking of a hidden hand and a visible rubber hand creates an ownership illusion of the fake hand (Botvinick and Cohen, 1998). These illusions manipulate sensory and motor cues to prompt ownership of artificial bodies, like mannequins (Botvinick and Cohen, 1998; Ehrsson et al., 2004, 2007; Tsakiris and Haggard, 2005; Tsakiris et al., 2006; Lloyd, 2007; Petkova and Ehrsson, 2008; Dummer et al., 2009; Guterstam et al., 2011; Kalckert and Ehrsson, 2012, 2014; Ide, 2013; Erro et al., 2020) or virtual avatars (Petkova and Ehrsson, 2008; Slater et al., 2009, 2010; Sanchez-Vives et al., 2010; Yuan and Steed, 2010; Kilteni et al., 2012; Won et al., 2015). In particular, they show that we can take ownership of a fake body that is in a different spatial location than our body (Botvinick and Cohen, 1998; Ehrsson et al., 2004, 2007; Blanke and Mohr, 2005; Tsakiris and Haggard, 2005; Lloyd, 2007; Riva et al., 2007; Petkova and Ehrsson, 2008; Dummer et al., 2009; Guterstam et al., 2011; Kalckert and Ehrsson, 2012, 2014; Ide, 2013; Kilteni et al., 2015; Erro et al., 2020). The illusion is possible so long as the location and orientation of the fake body part are anatomically plausible (Kilteni et al., 2015). Applying a rotation to the false body part in an anatomically implausible configuration, such as rotating the hand 180°, reduces the illusory experience (Ehrsson et al., 2004; Kalckert and Ehrsson, 2012; Ide, 2013). There is a significant drop in sensations of ownership when the location of the fake body part is far from the real body part (Lloyd, 2007; Sanchez-Vives et al., 2010; Erro et al., 2020) and beyond its anatomical reach. Last, the illusion does not occur with an anatomically incongruent fake body part (Graziano et al., 2000; Tsakiris and Haggard, 2005; e.g., a fake right-hand in a left-hand illusion), which defies the anatomical configuration of the joints.

Yet, participants in these RHI studies had limited interaction with their external environment. The experiments use passive touch (Tsakiris and Haggard, 2005; Lloyd, 2007; Guterstam et al., 2011; Ide, 2013; Kalckert and Ehrsson, 2014; Erro et al., 2020) or restrict actions to a narrow range of predetermined movements (Kalckert and Ehrsson, 2014) such as finger tapping with little goal-direct movement. These interactions consist of a narrow set of sensorimotor cues compared to the complex ways we use our body and take ownership of it. Although the RHI provides an easy and replicable way to study body ownership, there is a need for an ecological and realistic environment to examine anatomical plausibility constraints. In the current study, we use immersive virtual reality to challenge previous assumptions on anatomical plausibility. We chose virtual reality to precisely manipulate the illusion and control experimental conditions in a way that would hardly be possible in real life (Bohil et al., 2011).

**Figure 1:**
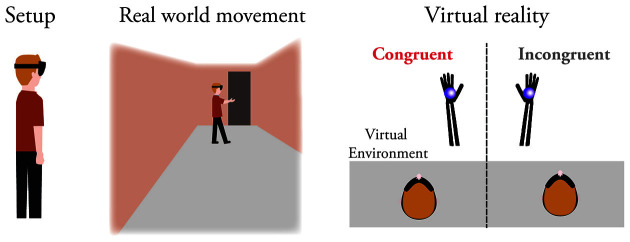
Experimental setup and virtual environment. Participants wear a head-mounted display with a sensor that tracks both hands. They can move freely within the room, using their hands to play with a virtual ball (right hand in the figure). In virtual reality, the avatar hands can be congruent with the real hands or switched and incongruent. The figure on the right shows the virtual display of a participant’s right arm in congruent and incongruent conditions. In each experimental condition, both hands were either congruent or incongruent.

In the virtual environment, hand movements were visually switched and mirrored. Hand movements result in visual feedback of the other hand’s analogous movements (Figure 1). We thus applied three anatomically implausible transformations to the fake hands—their location constantly changes and can be far from the real hands (distance constraint), they are at a wide-angle to the real hands (angle constraint), and their physical attributes are incongruent with the real hands (anatomical incongruency constraint). We developed two interactive playing scenarios where participants use their switched hands to hit and lift virtual balls in an office-like setting (Figure 1). In experiment 1, one group of participants performed the scenarios while their real hands were incongruent with the virtual hands. Another group participated in the same experiment while their real hands were congruent with the virtual hands. After the virtual reality, participants from both groups completed a questionnaire on their subjective sense of body ownership, agency, and self-location (Gonzalez-Franco and Peck, 2018). In experiment two, participants performed both the incongruent and congruent conditions (in random order). Participants answered the questionnaire at the end of each condition. We hypothesize that purposeful tasks in an immersive setting would increase the level of ownership towards the virtual avatar (Slater et al., 2009; Sanchez-Vives et al., 2010; Yuan and Steed, 2010; Won et al., 2015) even when there are vast anatomical discrepancies between the real and fake hands (Slater et al., 2010; Feuchtner and Müller, 2017). We further predict that sensations of ownership and agency will not depend on the perceived location of the avatar, similarly to previous studies (Kilteni et al., 2015; Gonzalez-Franco and Peck, 2018). In addition, such a result would demonstrate that the fake hand’s location does not have to follow strict anatomical constraints, as previously assumed (Kilteni et al., 2015).

## Materials and Methods

### Participants

A total of 49 healthy participants took part in experiment 1 (age 28.2 ± 7.2, average and standard deviation; 30 females; 49 right-hand dominants); 29 performed the virtual reality incongruent condition with switched hands, the other 20 performed the congruent condition (Figure 1). Another 20 more participants took part in experiment 2 (age 27.1 ± 5.4, average and standard deviation; six females; 20 right-hand dominants). We counterbalanced the condition order in experiment 2, with 10 participants starting with the congruent condition and 10 with the incongruent condition. All participants reported normal or corrected-to-normal vision with no known neurological deficits.

### Materials

We developed the virtual environment using the Unity 3D engine (Unity Technologies). We used the VIVE Pro head-mounted display (HTC Corporation) to convey the virtual environment (Figure 1) and a LeapMotion sensor (Ultrahaptics) to track participants’ hand gestures. To switch participants’ hands, we developed a real-time algorithm that receives the hands’ location from the sensor, transposes the hands’ coordinates, and displays the transposed figures as avatars. All the visual assets in VR are of our creation and made for this study.

### Procedure

We first instructed participants about the experiment and informed them, if needed, about the incongruent condition. The virtual environment is a 2.5 by 3 meters virtual office space with an ‘r-shaped’ desk and a blue curtain. In the congruent condition, participants view a virtual representation of their hands that overlaps with their real hands. In the incongruent condition, we switched participants’ hands. When participants move their hands, they see the opposite virtual hand moving (Figure 1). Participants in experiment 1 completed one condition, while participants in experiment 2 completed two conditions. Each condition includes two consecutive scenarios—(a) A bowl stands in the middle of the virtual desk with a single ball on each side. In each trial, the participant picks up a ball with one hand and places it in the bowl. In experiment 1, the scenario ends when the participant completes 16 successful tries or 5 min have elapsed. In experiment 2, the scenario ends after 3 min; (b) We remove the bowl while two balls remain on the desk. In each trial, the participant tries to push a ball off the desk following an auditory cue. The cue consists of instructions on which virtual hand to use (left or right) and the proceeding action (push the right or left ball). The scenario includes 40 trials in experiment 1 and 20 trials in experiment 2. The trials were equally divided between the four hand and ball combinations, with an inter-stimulus interval of 15 s. We consistently instructed participants to keep their hands separate to cut contradicting tactile and visual information, but otherwise, move freely within the space (Figure 1).

### Questionnaire

At the experiment conclusion, participants complete a questionnaire (Gonzalez-Franco and Peck, 2018) on their subjective sense of ownership (three questions), agency (four questions), and self-location (two questions). The questionnaire is particularly for VR experiments and builds on previous questions that appear in the literature. Participants scores each statement on a 7-point Likert scale that ranges from–3 (“strongly disagree) to 3 (“strongly agree”). Participants in experiment 2 filled the questionnaire twice, once at the end of each condition. A full description of the statements and ratings appears in Supplementary Table S1.

### Statistical Analyses

We summarized participants’ responses to a single score for ownership, self-location, and agency (Supplementary Table S2). Following on similar studies (Ehrsson et al., 2007; Petkova and Ehrsson, 2008; Guterstam et al., 2011; Kalckert and Ehrsson, 2012, 2014; Kilteni et al., 2012), we interpreted a group result as meaningful if the median score was equal to or higher than 1. We then conducted a one-way Wilcoxon-signed rank test on the median score. In experiment 1, we used a two-way Wilcoxson rank-sum test to analyze group differences in each category. We also used a two-way ANOVA to calculate the interaction effect of a category within-factor and a group between-factor (Supplementary Tables S3–S5). In experiment 2, we used a paired Wilcoxson signed-rank test to analyze the differences in questionnaire ratings between the congruent and incongruent conditions. We used a two-way ANOVA with a category within-factor and a condition within-factor (Supplementary Tables S6–S8). We then examined the effect of condition order (congruent first or incongruent first) on each category rating with a within-factor of condition and a between-factor of order (Supplementary Tables S9–S11). In both experiments, we calculated the Person correlation between body-ownership ratings and the other categories. We also conducted a mediation analysis to examine if sensations of agency or self-location mediate the effects of condition on body ownership (see Supplementary Table S12 and Supplementary Figure S1 for full details). All the statistical analyses included Bayes Factors inference calculations (Liang et al., 2008; Rouder et al., 2012; Faulkenberry, 2021). A Bayes Factor score below 3 is inconclusive, over 10 is strong, and over 100 is decisive (Lee and Wagenmakers, 2014). We conducted all the analyses using the MATLAB software (MathWorks), statistical tests were double-sided and corrected for multiple comparisons using False Discovery Rate (*α* = 0.05). Where the correction deemed the score insignificant, we also added a corrected p-value. Effect sizes in the Wilcoxon tests are Cliff’s Delta and Theta square in the ANOVA tests.

## Results

### Participants Report Ownership and Agency of Switched Hands

**Figure 2:**
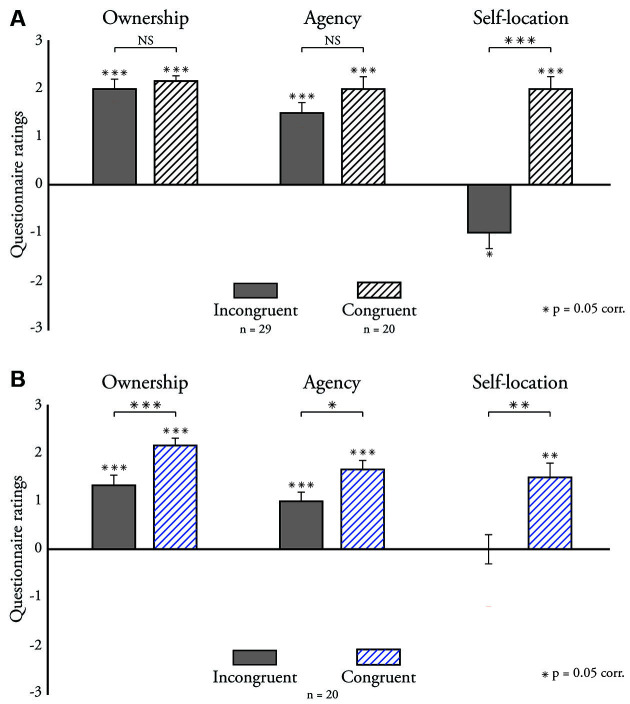
Median questionnaire ratings. **(A)** Experiment 1. We compared questionnaire ratings between the incongruent (*n* = 29) and congruent (*n* = 20) groups on body ownership, agency, and self-location. **(B)** Experiment 2. We measured and compared participants’ ratings of body ownership, agency, and self-location in the congruent and incongruent conditions (*n* = 20). Error bars indicate the standard error. *p < 0.05; **p < 0.005; ***p < 0.0005; NS, Not Significant.

We first analyzed the questionnaire ratings on ownership and agency (Figure 2). Experiment 1. Participants in the congruent group (*n* = 20) reported a strong sense of body ownership (*M* = 2 ± 0.21, *p* < 0.001, *W* = 210, δ = 1, BF10 > 100) and agency (*M* = 2 ± 0.26, *p* < 0.001, *W* = 184.5, δ = 0.85, BF10 = 52.9). We further found high ratings in the incongruent group (*n* = 29) for ownership (*M* = 2 ± 0.21, *p* < 0.001, *W* = 362, δ = 0.79, BF10 > 100) and agency (*M* = 1.5 ± 0.22, *p* < 0.001, *W* = 407.5, δ = 0.86, BF10 > 100). The rank-sum test showed no significant group differences that survived correction for multiple comparisons, neither for ownership (*Z* = 2.14, *p* = 0.034, *W* = 604, δ = 0.36, adjusted *p* = 0.102) nor agency scores (*Z* = 1.12, *p* = 0.262, *W* = 555.5, δ = 0.19). The Bayes Factors analysis further confirmed the group null results for agency ratings (BF10 = 0.24) and was inconclusive for ownership ratings (BF10 = 1.99). We next conducted an ANOVA test on the questionnaire scores with a with-in factor of the category (agency and ownership) and a between-factor of the group to compute an interaction effect on the factors (Supplementary Table S5). The interaction between the factors was insignificant (*F*_(1, 94)_ = 0.49, *p* = 0.488, *η*^2^ < 0.01, BF10 = 0.13), which indicates that switching hands did not alter the difference between agency and ownership (Figure 2A). Experiment 2. The within-group study (*n* = 20) replicated the main results from experiment 1 (Figure 2B). In the congruent condition, participants had a strong sense of ownership (*M* = 2.17 ± 0.15, *p* < 0.001, *W* = 210, δ = 1, BF10 > 100) and agency (*M* = 1.67 ± 0.19, *p* < 0.001, *W* = 210, δ = 1, BF10 >100). The ratings in the incongruent condition were also strong for ownership (*M* = 1.33 ± 0.21, *p* < 0.001, *W* = 165.5, δ = 0.8, BF10 = 31.6) and agency (*M* = 1 ± 0.19, *p* < 0.001, *W* = 169, δ = 0.8, BF10 = 58.6). Unlike experiment 1, we found significant differences between the congruent and incongruent conditions on ownership ratings (*Z* = 3.56, *p* < 0.001,*W* = 167, δ = 0.56, BF10 > 100) and agency ratings (*Z* = 2.69, *p* = 0.007, *W* = 147, δ = 0.38, BF10 = 10.7). Like experiment 1, an ANOVA analysis with two with-in factors of category and condition (Supplementary Table S8) showed no significant interaction on the factors (*F*_(1, 76)_ = 0.71, *p* = 0.401, *η*^2^ < 0.01, BF10 = 0.43).

### Virtual Switched Hands Are Not Perceived as Collocated With Real Hands

We analyzed participants’ reports on the self-location of the avatar in comparison to their real hands (Figure 2). Experiment 1. Participants in the congruent group (Figure 2A) reported that the virtual hands’ position corresponded to the location of their real hands in space (*M* = 2 ± 0.26, *p* < 0.001, *W* = 186, δ = 0.75, BF10 = 63.9). In contrast, participants in the incongruent group (Figure 2A) perceived that the virtual hands’ location did not correspond with their real hands (*M* = −1 ± 0.34, *p* = 0.015, *W* = 73.5, δ = −0.31, BF10 = 3.2). An analysis of group differences shows that location ratings were higher in the congruent group (*Z* = 4.57, *p* < 0.001, *W* = 723, δ = 0.77, BF10 > 100). We performed an ANOVA to observe the interaction effect of condition with category ratings of self-location and ownership, or self-location and agency (Supplementary Tables S3, S4). There was a significant interaction effect of self-location with the agency (*F*_(1, 94)_ = 17.11, *p* < 0.001, *η*^2^ = 0.11, BF10 > 100) and self-location with body ownership (*F*_(1, 94)_ = 15.06, *p* < 0.001, *η*^2^ = 0.08, BF10 > 100). Experiment 2. Self-location ratings in the within-group experiment corroborated the results of experiment 1 (Figure 2B). In the congruent condition, participants had a strong sense of self-location (*M* = 0 ± 0.31, *p* = 0.869, *W* = 49.5, δ = 0.1, BF10 = 0.43), while self-location ratings in the incongruent condition were weak (*M* = 1.5 ± 0.3, *p* = 0.001, *W* = 158, δ = 0.5, BF10 = 19). A paired analysis showed higher self-location ratings in the congruent condition (*Z* = 3.26, *p* = 0.001, *W* = 160, δ = 0.5, BF10 > 100). Unlike experiment 1, the ANOVA analyses (Supplementary Tables S6, S7) did not show significant interaction effects on ratings of self-location with the agency (*F*_(1, 76)_ = 2.61, *p* = 0.111, *η*^2^ = 0.02, BF10 = 0.43) and self-location with ownership (*F*_(1, 76)_ = 0.99, *p* = 0.324, *η*^2^ = 0.01, BF10 = 0.16).

### Condition Order Did Not Affect Ratings of Ownership, Agency, or Self-location

Experiment 2. We explored the effects of starting the experiment in the congruent (*n* = 10) or incongruent (*n* = 10) condition on questionnaire ratings. We conducted a three two-way ANOVA (Supplementary Tables S9–S11), one for each category rating, with a within-factor of condition (congruent/incongruent) and a between-factor of order (congruent first/incongruent first). The results were not significant for the main effect of condition order in self-location ratings (*F*_(1, 36)_ = 0.01, *p* = 0.91, *η*^2^ < 0.01, BF10 = 0.16), agency (*F*_(1, 36)_ = 0.46, *p* = 0.503, *η*^2^ = 0.01, BF10 = 0.2), and body ownership (*F*_(1, 36)_ = 0.25, *p* = 0.623, *η*^2^ < 0.01, BF10 = 0.18). We also did not find any interaction effects on the factors for self-location (*F*_(1, 36)_ = 0.12, *p* = 0.735, *η*^2^ < 0.01, BF10 = 0.17), agency (*F*_(1, 36)_ = 1.09, *p* = 0.303, *η*^2^ = 0.03, BF10 = 0.29), or ownership (*F*_(1, 36)_ = 0.02, *p* = 0.902, *η*^2^ < 0.01, BF10 = 0.16).

### Switched Hands’ Effect on Body Ownership Is Mediated by Agency, but Not by Self-location

*Experiment 1*. Self-location did not correlate with ownership in the congruent group (*R* = 0.17, *Z* = 0.7, *p* = 0.486, BF10 = 0.22) nor the incongruent group (*R* = −0.19, *Z* = 0.96, *p* = 0.335, BF10 = 0.22). Agency and ownership did not correlate in the congruent group (*R* = 0.21, *Z* = 0.9, *p* = 0.375, BF10 = 0.25) but correlated in the incongruent group (*R* = 0.6, *Z* = 3.44, *p* < 0.001, BF10 = 50.96). We found that the condition can affect body ownership ratings (*t*(*β*_1_) = 2.38, *p*(*β*_1_) = 0.021, *R^2^* = 0.11, BF10 = 1.55). But, the agency does not mediate the effect (Supplementary Table S13), nor is the effect mediated by self-location (Supplementary Table S14). *Experiment 2*. Self-location correlated with ownership in the congruent condition (*R* = 0.57, *Z* = 2.6, *p* = 0.009, BF10 = 4.87) but did not in the incongruent condition (*R* = −0.01, *Z* = 0.1, *p* = 0.921, BF10 = 0.17). Agency did not correlate with ownership in the congruent condition (*R* = 0.362, *Z* = 1.57, *p* = 0.117, BF10 = 0.58) nor the incongruent condition (*R* = 0.39, *Z* = 1.72, *p* = 0.086, BF10 = 0.74). In the mediation analysis, we found that condition affects body ownership ratings (*t*(*β*_1_) = 3.42, *p*(*β*_1_) = 0.002, *R^2^* = 0.24, BF10 = 18.28). Self-location does not mediate the effect (Supplementary Table S16), but the effect is partially mediated by the agency (Supplementary Table S15). When controlling for condition (*β*_1_), agency (*β*_2_) still showed a significant effect on body ownership (*t*(*β*_1_) = 2.62, *p*(*β*_1_) = 0.013, *t*(*β_2_*) = 2.47, *p*(*β*_1_) = 0.018, *R^2^* = 0.31, BF10 = 19.01).

## Discussion

The current study explored the anatomical plausibility constraints of body ownership illusions. We used virtual reality to develop two immersive environments where participants’ fake hands are incongruent or congruent with their real hands. In our between-group experiment, participants reported a strong sense of body ownership in the congruent condition, confirming the immersive properties of the virtual environment (Riva et al., 2007; Petkova and Ehrsson, 2008; Slater et al., 2009, 2010; Sanchez-Vives et al., 2010; Yuan and Steed, 2010; Kilteni et al., 2012; Kuliga et al., 2015; Feuchtner and Müller, 2017). We also found that participants in the incongruent group had a strong sense of body ownership, despite the hand switch.

We replicated these results in our within-group experiment that included condition conditions. Participants reported a strong sense of body ownership in the incongruent condition despite the fact they also experienced the congruent condition. These findings contradict previous assumptions that body ownership illusions are contingent on the anatomical plausibility of the fake body part (Graziano et al., 2000; Ehrsson et al., 2004; Tsakiris and Haggard, 2005; Lloyd, 2007; Sanchez-Vives et al., 2010; Kalckert and Ehrsson, 2012; Ide, 2013; Erro et al., 2020). Our setup forms an extreme instance of anatomical implausibility that violates its three known constraints. Participants performed manual tasks with virtual avatars of the opposite and incongruent hands (Tsakiris and Haggard, 2005) whose locations are distant from (Lloyd, 2007; Sanchez-Vives et al., 2010; Erro et al., 2020) and at an angle to their real hands (Ehrsson et al., 2004; Kalckert and Ehrsson, 2012; Ide, 2013). Contrary to a prediction of failed ownership illusion under such conditions, we found that participants report significant sensations of ownership over the anatomically implausible hands. We propose that this finding links to goal-directed tasks undertaken by our participants that resulted in increased feelings of body agency.

Agency is the sense of intending and executing actions, such as the feeling of controlling one’s voluntary movements and their effects on the external environment (Tsakiris et al., 2006). The sense of agency is not uniform and includes multiple, perhaps separate, processes. For instance, we can experience agency over an external object in disassociation from our body (external agency), such as controlling an avatar in a computer game. We can also have agency over our somatic actions (body agency), like the purposeful movement of our hands (Kalckert and Ehrsson, 2012). Though agency and body ownership are somewhat disassociated (Kalckert and Ehrsson, 2012; Braun et al., 2018), this type of “body agency” can promote feelings of ownership if present (Kalckert and Ehrsson, 2012). Body agency could thus boost the sensations of body ownership our participants report in the incongruent condition. Yet, it is unclear what experimental and sensorimotor circumstances can bring about body agency rather than an external agency. Participants in previous studies on anatomical implausibility had reported low levels of body ownership coupled with high levels of agency (Tsakiris and Haggard, 2005; Lloyd, 2007; Guterstam et al., 2011; Ide, 2013; Kalckert and Ehrsson, 2014; Erro et al., 2020). The discrepancy in sensations may be due to the limited and inconsequential tasks that participants execute (Kalckert and Ehrsson, 2012). We propose that agency and ownership sensations reported in our study rest on goal-directed and meaningful actions in the form of affordances (Gibson, 1977). According to this theory, tasks of increasing complexity and unpredictability promote sensations of body ownership (Van Den Bos and Jeannerod, 2002; Kilteni et al., 2015). The complex interplay between body ownership and agency could be the subject of a future study where the manual task and its purposefulness are independent variables.

In experiment 1, we did not find any interaction between agency and body ownership ratings in the group analysis, which fits the non-significant differences in individual category ratings. On the other hand, we found that participants in experiment 2 reported weaker sensations of ownership and agency over the switched hands. Surprisingly, we again did not find any interaction on the categories when comparing between conditions. Further analysis revealed that agency mediates the effect of hand congruency on body ownership ratings. Although the virtual scenario is similar in both experiments, the context of the experience changes the relationship between agency and ownership. When participants can compare the experiences of both conditions, they report weaker sensations of agency and body ownership. A possible explanatory factor is the shortened time duration participants spent in each virtual scenario compared to experiment 1. Sensations of agency take time to emerge and follow a temporal learning curve shared by infants and adults alike (Haggard, 2017). In our case, participants might take longer to gain control over the incongruent hands that, in turn, leads to weaker sensations of ownership.

Like previous studies, we find that participants report sensations of ownership even when the fake hands are not collocated with their hands (Kilteni et al., 2015; Gonzalez-Franco and Peck, 2018). Yet, this finding contradicts the assumption that body ownership illusions are contingent on the proximity of the fake hand, which must be in reach of the physical hand (Lloyd, 2007; Sanchez-Vives et al., 2010; Erro et al., 2020). We observe that, under certain conditions, a greater distance between the real and fake hands does not cancel the perception of body ownership.

Our findings also show the capabilities of virtual reality as an effective platform to create subjective experiences that would not otherwise be possible. Virtual reality allows for detailed observations, accurate behavioral measurements, and systematic environmental manipulations under controlled laboratory conditions (Blascovich et al., 2002; Kuliga et al., 2015). More immersive systems can produce higher levels of behavioral realism (Slater et al., 2006), where the user experiences the environment as if it was part of the real world. In conclusion, the present study challenges previous assumptions and shows that body ownership illusions can extend to fake body parts that are anatomically implausible.

## Data Availability Statement

The raw data supporting the conclusions of this article will be made available by the authors, without undue reservation.

## Ethics Statement

The studies involving human participants were reviewed and approved by The Interdisciplinary Center Herzliya (IDC), School of Psychology ethics committee. The patients/participants provided their written informed consent to participate in this study.

## Author Contributions

OY drafted the manuscript. JG, MW, DC, DF, and AA provided revisions and approved the draft for submission. The virtual environment was developed by JG, MW, and GO with help from DF. OY, MW, and DC collected the data. OY analyzed the data, interpreted the results, and produced all the figures. AA provided the funding for the study. All authors contributed to the article and approved the submitted version.

## Conflict of Interest

The authors declare that the research was conducted in the absence of any commercial or financial relationships that could be construed as a potential conflict of interest.

## Publisher’s Note

All claims expressed in this article are solely those of the authors and do not necessarily represent those of their affiliated organizations, or those of the publisher, the editors and the reviewers. Any product that may be evaluated in this article, or claim that may be made by its manufacturer, is not guaranteed or endorsed by the publisher.
