# Supplementary material for: Body Ownership of Anatomically Implausible Hands in Virtual Reality
Source: Front Hum Neurosci. 2021 Nov 3;15:713931. doi: 10.3389/fnhum.2021.713931 (PMC8595134; doi:10.3389/fnhum.2021.713931)
Supplement: Supplementary file 1 [file Data_Sheet_1.PDF]

Body Ownership of Anatomically Implausible Hands in Virtual Reality

Or Yizhar*[1,2], Jonathan Giron[3], Mohr Wenger[1,2], Debbie Chetrit[4], Gilad Ostrin[4], Doron Friedman[4], Amir Amedi[2,5]

[1]Department of Cognitive and Brain Sciences, The Hebrew University of Jerusalem, Jerusalem, Israel, [2]Baruch Ivcher School of Psychology, Interdisciplinary Center Herzliya, Herzliya, Israel, [3]Innovation Center, Interdisciplinary Center Herzliya, Herzliya, Israel, [4]Sammy Ofer School of Communications, Interdisciplinary Center Herzliya, Herzliya, Israel, [5]The Ruth & Meir Rosental Brain Imaging Center, Interdisciplinary Center Herzliya, Herzliya, Israel

* Corresponding author: or.yizhar@mail.huji.ac.il

SUPPLEMENTARY MATERIAL

SUPPLEMENTARY TABLES AND FIGURES

Table S1. Standardized questionnaire completed by participants and the end of the experiment.

Please read the following statements and respond according to your experience in the virtual environment. Each statement is rated on a scale between "strongly disagree" and "strongly agree".

	**Strongly disagree**	**Disagree**	**Somewhat disagree**	**Neutral**	**Somewhat agree**	**Agree**	**Strongly agree**
**Q1**: I felt as if the virtual hands were my hand	○	○	○	○	○	○	○
**Q2**: It felt as if the virtual hands I saw were someone else's	○	○	○	○	○	○	○
**Q3**: It felt as if I have more than 2 hands	○	○	○	○	○	○	○
**Q4**: It felt like I could control the virtual hands as if they were my own hands	○	○	○	○	○	○	○
**Q5:** The movements of the virtual hands were caused by my movements	○	○	○	○	○	○	○
**Q6:** I felt as if the movements of the virtual hands were influencing my own movements	○	○	○	○	○	○	○
**Q7**: I felt as if the virtual hands were moving by themselves	○	○	○	○	○	○	○
**Q8**: I felt out of my body	○	○	○	○	○	○	○
**Q9**: I felt as if my hands were located where I saw the virtual hands	○	○	○	○	○	○	○

Table S2. Calculation of category scores from questionnaire.

Category	Formula
Ownership	(Q1 − Q2 − Q3) / 3
Agency	(Q4 + Q5 – Q6 – Q7) / 4
Self-location	(Q9 – Q8) / 2

Table S3. *Experiment 1.* Two-way ANOVA with a within-factor of category (self-location, agency) and a between-factor of group condition (congruent, incongruent).

	SS	*df*	*F*	*P-value*	*Eta squared*
Category	28.1	1	14.66	<0.001	0.09
Group	53.9	1	28.13	<0.001	0.18
Interaction	32.8	1	17.11	<0.001	0.11
Error	180.3	94			

Table S4. *Experiment 1.* Two-way ANOVA with a within-factor of category (self-location, ownership) and a between-factor of group condition (congruent, incongruent).

	SS	*df*	*F*	*P-value*	*Eta squared*
Category	47.7	1	28.73	<0.001	0.15
Group	65.2	1	39.22	<0.001	0.21
Interaction	25.0	1	15.06	<0.001	0.08
Error	156.2	94			

Table S5. *Experiment 1.* Two-way ANOVA with a within-factor of category (agency, ownership) and a between-factor of group condition (congruent, incongruent).

	SS	*df*	*F*	*P-value*	*Eta squared*
Category	2.6	1	2.37	0.127	0.02
Group	5.5	1	5.05	0.027	0.05
Interaction	0.5	1	0.49	0.488	<0.01
Error	102.3	94			

Table S6. *Experiment 2.* Two-way ANOVA with a within-factor of category (self-location, agency) and a within-factor of condition (congruent, incongruent).

	SS	*df*	*F*	*P-value*	*Eta squared*
Category	16.5	1	12.85	<0.001	0.12
Condition	19.7	1	15.32	<0.001	0.14
Interaction	3.3	1	2.61	0.111	0.02
Error	97.5	76			

Table S7. *Experiment 2.* Two-way ANOVA with a within-factor of category (self-location, ownership) and a within-factor of condition (congruent, incongruent).

	SS	*df*	*F*	*P-value*	*Eta squared*
Category	26.4	1	20.91	<0.001	0.18
Condition	26.5	1	20.92	<0.001	0.18
Interaction	1.2	1	0.99	0.324	<0.01
Error	96.1	76			

Table S8. *Experiment 2.* Two-way ANOVA with a within-factor of category (agency, ownership) and a within-factor of condition (congruent, incongruent).

	SS	*df*	*F*	*P-value*	*Eta squared*
Category	1.2	1	1.65	0.203	0.02
Condition	11.0	1	15.48	<0.001	0.16
Interaction	0.5	1	0.71	0.401	<0.01
Error	96.1	76			

Table S9. *Experiment 2.* Two-way ANOVA on self-location ratings with a between-factor of order (congruent first, incongruent first) and a within-factor of condition (congruent, incongruent).

	SS	*df*	*F*	*P-value*	*Eta squared*
Order	0.0	1	0.01	0.910	<0.01
Condition	19.6	1	10.15	0.003	0.22
Interaction	0.2	1	0.12	0.735	<0.01
Error	69.6	36			

Table S10. *Experiment 2*. Two-way ANOVA on agency ratings with a between-factor of group order (congruent first, incongruent first) and a within-factor of condition (congruent, incongruent).

	SS	*df*	*F*	*P-value*	*Eta squared*
Order	0.3	1	0.46	0.503	0.01
Condition	3.4	1	4.60	0.039	0.11
Interaction	0.8	1	1.09	0.303	0.03
Error	26.5	36			

Table S11. *Experiment 2*. Two-way ANOVA on ownership ratings with a between-factor of group order (congruent first, incongruent first) and a within-factor of condition (congruent, incongruent).

	SS	*df*	*F*	*P-value*	*Eta squared*
Order	0.2	1	0.25	0.623	<0.01
Condition	8.1	1	11.18	0.002	0.24
Interaction	0.0	1	0.02	0.902	<0.01
Error	26.1	36			

Table S12. We hypothesized a causal link where independent variable X affects the outcome of Y through a mediation of variable M. We tested the mediation through a series of 4 regressions (Fig. S1). In step (4), some mediation exists if the effect of predictor M is still significant after controlling for the effect of X. If M is significant and X is not, there is a full mediation. If both X and M are significant there is a partial mediation.

(1)	Regression with X predicting Y	$Y = \beta_0 + \beta_1 X + \varepsilon$
(2)	Regression with X predicting M	$M = \beta_0 + \beta_1 X + \varepsilon$
(3)	Regression with M predicting Y	$Y = \beta_0 + \beta_1 M + \varepsilon$
(4)	If and only if (1)-(3) are significant, conduct multiple regression with X and M predicting Y	$Y = \beta_0 + \beta_1 X + \beta_2 M + \varepsilon$

Figure S1. Graphical depiction of mediation analysis.

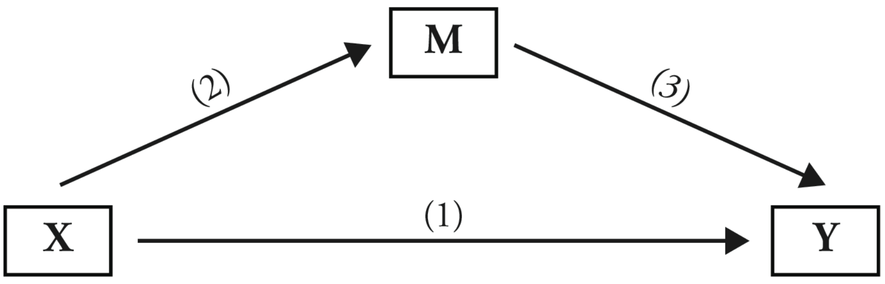

Table S13. *Experiment 1*. Mediation of Agency (M) on the effect of Condition (X) on Body Ownership (Y).

	(1)		(2)		(3)	
	β_0	β_1	β_0	β_1	β_0	β_1
Coefficient	1.59	0.63	0.22	0.38	0.2	0.1
t-stat	9.36	2.38	6.52	0.99	6.04	3.95
p	<0.001	0.021	<0.001	**0.329**	<0.001	<0.001
R^2	0.108		**0.02**		0.25	

Table S14. *Experiment 1.* Mediation of Self-location (M) on the effect of Condition (X) on Body Ownership (Y).

	(1)		(2)		(3)	
	β_0	β_1	β_0	β_1	β_0	β_1
Coefficient	1.58	0.63	-0.86	2.69	1.83	0.058
t-stat	9.36	2.38	-2.94	5.86	13.28	0.87
p	<0.001	0.021	0.005	<0.001	<0.001	**0.39**
R^2	0.108		0.422		0.016	

Table S15. *Experiment 2*. Mediation of Agency (M) on the effect of Condition (X) on Body Ownership (Y).

	(1)		(2)		(3)		(4)		
	β_0	β_1	β_0	β_1	β_0	β_1	β_0	β_1	β_2
Coefficient	1.25	0.9	1.17	0.58	0.98	0.49	0.82	0.69	0.37
t-stat	6.72	3.42	6.11	2.16	3.8	3.29	3.34	2.62	2.47
p	<0.001	0.002	<0.001	0.03	<0.001	0.002	0.002	0.013	0.018
R^2	0.236		0.109		0.2214		0.308		

Table S16. *Experiment 2.* Mediation of Self-location (M) on the effect of Condition (X) on Body Ownership (Y).

	(1)		(2)		(3)		(4)		
	β_0	β_1	β_0	β_1	β_0	β_1	β_0	β_1	β_2
Coefficient	1.25	0.90	-0.15	1.40	1.56	0.25	1.27	0.71	0.13
t-stat	6.72	3.42	-0.49	3.27	10.63	2.66	6.88	2.42	1.36
p	<0.001	0.002	0.623	0.002	<0.001	0.011	<0.001	0.021	**0.182**
R^2	0.236		0.219		0.157		0.272		
